# Harnessing Machine Learning in Early COVID-19 Detection and Prognosis: A Comprehensive Systematic Review

**DOI:** 10.7759/cureus.38373

**Published:** 2023-05-01

**Authors:** Rufaidah Dabbagh, Amr Jamal, Jakir Hossain Bhuiyan Masud, Maher A. Titi, Yasser S Amer, Afnan Khayat, Taha S Alhazmi, Layal Hneiny, Fatmah A. Baothman, Metab Alkubeyyer, Samina A. Khan, Mohamad-Hani Temsah

**Affiliations:** 1 Family & Community Medicine Department, College of Medicine, King Saud University, Riyadh, SAU; 2 Research Chair for Evidence-Based Health Care and Knowledge Translation, Family and Community Medicine Department, College of Medicine, King Saud University, Riyadh, SAU; 3 Health Informatics, Public Health Informatics Foundation, Dhaka, BGD; 4 Quality Management Department, King Saud University Medical City, Riyadh, SAU; 5 Pediatrics, Quality Management Department, King Saud University Medical City, Riyadh, SAU; 6 Health Information Management Department, Prince Sultan Military College of Health Sciences, Al Dhahran, SAU; 7 Medicine, Wegner Health Sciences Library, University of South Dakota, Vermillion, USA; 8 Department of Information Systems, King Abdulaziz University, Jeddah, SAU; 9 Department of Radiology, King Saud University, Riyadh, SAU; 10 School of Computer Sciences, Universiti Sains Malaysia, Penang, MYS; 11 Pediatric Intensive Care Unit, Department of Pediatrics, King Saud University, Riyadh, SAU

**Keywords:** healthcare technology, deep learning artificial intelligence, covid-19 chest imaging, decision support systems, covid-19 diagnosis, prediction, artificial intelligence, machine learning in early pandemic, sars-cov-2, covid-19

## Abstract

During the early phase of the COVID-19 pandemic, reverse transcriptase-polymerase chain reaction (RT-PCR) testing faced limitations, prompting the exploration of machine learning (ML) alternatives for diagnosis and prognosis. Providing a comprehensive appraisal of such decision support systems and their use in COVID-19 management can aid the medical community in making informed decisions during the risk assessment of their patients, especially in low-resource settings. Therefore, the objective of this study was to systematically review the studies that predicted the diagnosis of COVID-19 or the severity of the disease using ML.

Following the Preferred Reporting Items for Systematic Reviews and Meta-Analysis (PRISMA), we conducted a literature search of MEDLINE (OVID), Scopus, EMBASE, and IEEE Xplore from January 1 to June 31, 2020. The outcomes were COVID-19 diagnosis or prognostic measures such as death, need for mechanical ventilation, admission, and acute respiratory distress syndrome. We included peer-reviewed observational studies, clinical trials, research letters, case series, and reports. We extracted data about the study's country, setting, sample size, data source, dataset, diagnostic or prognostic outcomes, prediction measures, type of ML model, and measures of diagnostic accuracy. Bias was assessed using the Prediction model Risk Of Bias ASsessment Tool (PROBAST). This study was registered in the International Prospective Register of Systematic Reviews (PROSPERO), with the number CRD42020197109.

The final records included for data extraction were 66. Forty-three (64%) studies used secondary data. The majority of studies were from Chinese authors (30%). Most of the literature (79%) relied on chest imaging for prediction, while the remainder used various laboratory indicators, including hematological, biochemical, and immunological markers. Thirteen studies explored predicting COVID-19 severity, while the rest predicted diagnosis. Seventy percent of the articles used deep learning models, while 30% used traditional ML algorithms. Most studies reported high sensitivity, specificity, and accuracy for the ML models (exceeding 90%). The overall concern about the risk of bias was "unclear" in 56% of the studies. This was mainly due to concerns about selection bias.

ML may help identify COVID-19 patients in the early phase of the pandemic, particularly in the context of chest imaging. Although these studies reflect that these ML models exhibit high accuracy, the novelty of these models and the biases in dataset selection make using them as a replacement for the clinicians' cognitive decision-making questionable. Continued research is needed to enhance the robustness and reliability of ML systems in COVID-19 diagnosis and prognosis.

## Introduction and background

Machine learning (ML), one of the broad disciplines of artificial intelligence (AI), refers to the ability of a machine to understand and learn hidden knowledge by finding patterns in large datasets using analytical techniques [1]. ML requires modeling design, learning functions, and developing algorithms. The main idea is to enable automated classification or clustering techniques to increasingly learn the behavior from data to generate new patterns and predict future actions using decision support systems [1]. Generally, ML can be broadly divided into three types: supervised learning, unsupervised learning, and reinforced learning [2]. The "supervised" method is the type often used in disease prediction. Supervised ML includes several classes such as regression, support vector machine, decision tree, random forest, naive Bayes, K-nearest neighborhood, and artificial neural network [3]. A more complex form of the neural network is deep learning (DL), which employs multiple layers of neural networks [4]. DL can be supervised, unsupervised, or reinforced.

ML has been frequently adopted as an aid for diagnostic screening during the COVID-19 pandemic, where the research suggests its ability to identify infected individuals from radiological imaging before symptoms develop [5]. ML technology also has the ability to process hundreds of thousands of images in a short period while exhibiting higher sensitivity and specificity for picking up radiological changes compared to the naked human eye [5]. At the beginning of the COVID-19 pandemic in 2020, there was an urgency to expand on what has been published concerning the operational maturity of ML as an aid for diagnosis and prognosis in the healthcare setting [6]. Some researchers express skepticism about the readiness of ML for deployment in COVID-19 prevention and control, given the limited scope and relatively poor quality of evidence in this area [7]. More importantly, while many ML models show good performance, they are at a very high risk of bias due to the limitation and non-representativeness of data samples, the selectivity of databases used for their development, and the lack of data access for model validation [8]. Thus, the literature shows a need for improvement to facilitate the safe and effective clinical adoption of ML applications during such a pandemic crisis [7]. Although many efforts have been made to use ML technology as a support system for COVID-19 in the clinical setting, the predictors and type of models used are very variable in nature, making it difficult for clinicians to evaluate the strengths and limitations of each. Several reviews have been published around the utility of ML technology to aid in the prediction of diagnosis and prognosis of COVID-19. However, these have their shortcomings. For starters, most of these reviews only studied the literature pertaining to using ML in diagnostic imaging [9-15].

On the other hand, others were too broad and included any use of AI in combating COVID-19 [16-18]. Few provided detailed summaries for the ML model types [19]. Some did not follow a methodologically sound systematic review approach [20,21], while others did not address bias assessment [21,22]. None provided variation in training and testing methods or the limitations of the datasets on which the models were developed and their applicability to the population in question. Moreover, due to the high demand for COVID-19 research, the previous reviews included many studies that have not yet been peer-reviewed [8,13,15].

Furthermore, many available reviews require technical expertise in ML, leaving technically inexperienced healthcare professionals in the dark. These challenges, among others, show that adopting ML models for the clinical setting should be approached with caution. Critical appraisal of such research needs to be critically appraised using a methodologically sound approach to help inform healthcare professionals. From that end, the aim of the current systematic review was two-fold. First, we summarized the literature published in the initial phase of the COVID-19 pandemic (January 1, 2020, to June 31, 2020) with respect to studies examining AI models for the prediction of diagnosis or prognosis of COVID-19. Second, we discussed the different model types, data sources, and diagnostic accuracy measures reported in these studies. With this review, we hope to bridge the gap between the ML technical savvy and lay medical readers.

## Review

Methods and materials

Search Strategy

We searched MEDLINE (OVID), Scopus, Embase, and IEEE Xplore, from the beginning of January to the end of June 2020, for all published studies that used ML models to predict the diagnosis or prognosis of COVID-19 using search string (Table 1).

**Table 1:** Set of search strings adapted to each of the databases searched

Search Keywords String
((((“pneumonia”) OR (“virus”) AND (“epidemiology”) OR (“outbreak” OR “wuhan” ) ) OR (“betacoronavirus”) OR (“beta-coronavirus” OR “coronavirus”) OR “covid” OR (“coronaviridae” ))) AND “Machine Learning” OR “knowledge W/2 ( acquisition* OR representation ) ) OR ( ( automated OR computat* OR artificial OR ambient ) W/2 ( intelligence OR reasoning ) ) OR ( comp uter W/1 ( reasoning OR ( vision-system ) ) ) OR ( ( data OR computational ) OR ((transfer OR m achine OR deep OR hierarchical OR supervised OR ( semi-supervised ) OR active OR inductive OR unsupervised ) W/1 learning ) OR (machine )OR network* ) ) OR ( clinical W/0 decision W/0 support* ) OR( ( ( augmented OR virtual ) W/0 reality ))))

LH conducted a database search, and results were exported to Endnote [23] to facilitate the collaboration of reviewers during the study selection process. The search strategy followed two stages and was conducted by the Preferred Reporting Items for Systematic Reviews and Meta-Analyses (PRISMA) of Observational Studies in Epidemiology reporting guidelines [24]. In the first stage, four investigators (RD, AJ, JH, and HT) independently screened the titles and abstracts of all the articles retrieved from the searched databases. If sufficient information was available in the abstract of an article to decide whether to retain or exclude it, the decision was made to exclude such articles from the full-text screening stage. Otherwise, the articles with titles relevant to the topic of interest, in which abstracts did not provide sufficient information for exclusion, were included in the full-text screening stage. During the second stage, the same four investigators screened the full text of all articles retained from the first stage for inclusion and exclusion criteria. When in doubt, disagreements were resolved with consensus.

Inclusion and Exclusion Criteria

We included observational studies, clinical trials, research letters, case series, and case reports addressing ML models in COVID-19 prediction without language restrictions. However, inclusion was restricted to articles that met the following criteria: (1) The article was published in a peer-reviewed journal; (2) the population was any patients with suspected SARS-CoV-2 infection or with a confirmed diagnosis when the prognosis was predicted; (3) the use of ML models was for assisting diagnosis or prognosis of suspected or diagnosed COVID-19 patients; and (4) the outcome of interest was COVID-19 diagnosis. We excluded time series, surveillance studies forecasting the COVID-19 pandemic, systematic or narrative reviews, opinions, short communications, commentaries, statement articles, news reports, preprints, and articles where we failed to access full text despite contacting the authors. However, preprints that were published at the time of writing this article were included. We also excluded any study that only used ML models to predict the diagnosis or prognosis of diseases other than COVID-19 or studies that predicted the diagnosis or prognosis of COVID-19 without ML. Two authors (RD and MA) resolved the discrepancies through discussion and adjudication.

As the influx of publications was very high during the early period of the pandemic and journal review processes were hastened, many articles published early in the pandemic may not have been reviewed stringently and were retracted later. For this reason, we made a final check on our retrieved articles and excluded any rejected ones when submitting this article.

Data Extraction

Data for each of the included articles were extracted by any of the two authors independently (RD, JH, MT, AJ, HT, MA, AK, SAK, and TA). A calibration exercise was conducted to ensure reviewers' consistency before the data extraction. The consensus of three authors (RD, JH, and MA) resolved any discrepancies in data extraction. The extracted measures included the first author’s name, author’s country, study field (radiology vs. other), study setting (hospital vs. computer lab), type of data source (primary vs. secondary), source database, sample size (total, training, and testing), the reported purpose of study, diagnostic outcomes of interest, predictive effects of interest, type of ML model used, and tests for diagnostic accuracy registered.

Bias Assessment

As most of the retrieved studies tested the performance of ML models for diagnostic or prognostic accuracy, we assessed the risk of bias using the Prediction model Risk Of Bias ASsessment Tool (PROBAST) [25]. The same authors who extracted the data also evaluated the risk of bias for their same assigned studies. Two authors (RD and MT) reviewed their assessments and checked the overall study ratings.

Data Synthesis

In this study, we provided a descriptive summary of the extracted data points and an overall rating for bias risk. Due to the high heterogeneity of ML models between studies and variation in sample populations and tests of diagnostic accuracy, it was not suitable to synthesize pooled accuracy estimates. This systematic review was registered on the International Prospective Register for Systematic Reviews (PROSPERO), with the number CRD42020197109 [26].

Results

**Figure 1:**
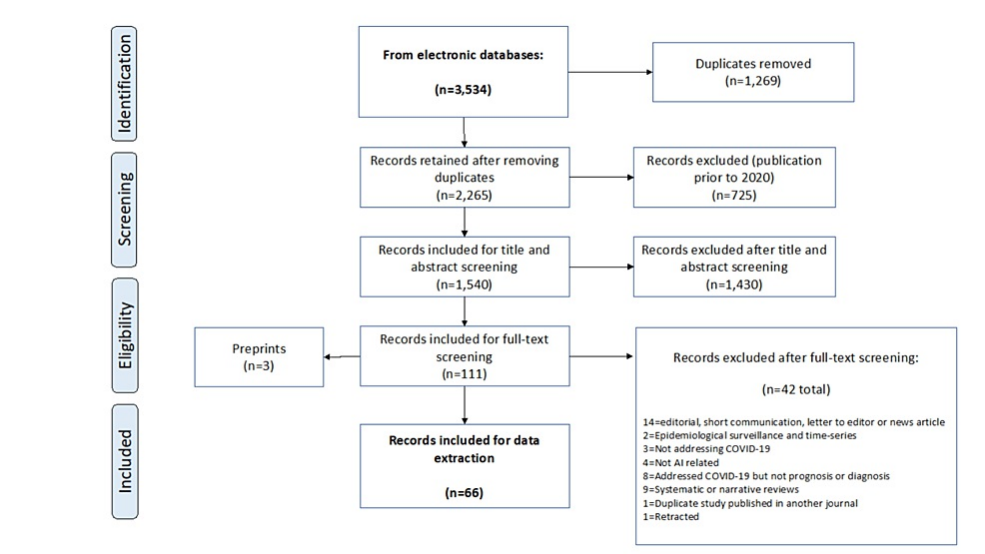
PRISMA chart for retrieval of included articles PRISMA: Preferred Reporting Items for Systematic Reviews and Meta-Analyses.

We retrieved 3,534 studies from the electronically searched databases, of which 110 were finally included for full-text screening (Figure 1).

Forty-one records were excluded from the data extraction stage because they met the exclusion criteria. Of these, three were still preprints at the time of writing this article. Additionally, a duplicate study published the same results in another journal. The final records from which data were extracted were 66 [27-94].

Characteristics of the Included Studies

**Figure 2:**
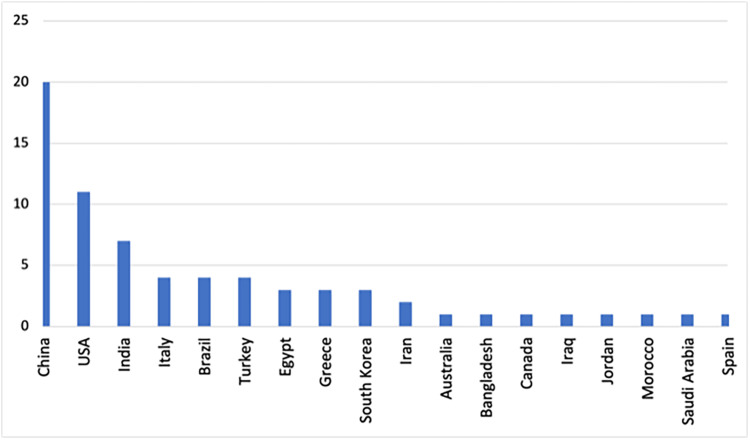
Number of publications by country based on authors' countries

Most retrieved publications were from Chinese authors (30%) (Figure 2).

Overall, 42 (64%) studies used publicly available secondary data (Appendix 1). The most commonly used source for COVID-19 radiographic images was the Joseph Cohen dataset [95], while the most frequent source for non-COVID-19 radiographic images was Kaggle.com** **[96]. Although these databases were frequently used in the included studies, there was insufficient information to evaluate the similarity of samples retrieved from these publicly available data. As a result of having these available secondary data sources for COVID-19 cases and non-COVID-19 individuals, the most common study design was an un-nested case-control design (71%). Of the studies that used primary hospital data (29), the data were predominantly from Chinese hospitals (19), most of which were from Wuhan province. Few studies had smaller sample sizes of fewer than 100 patients [27,44,46,48,51,91]. In one study, the sample was not mentioned altogether [66]. An important observation was that 52 studies (79%) were in the field of radiology in which the ML models were developed using chest radiographs for predicting COVID-19 diagnosis and distinguishing it from other lung diseases or predicting disease severity among hospitalized COVID-19 individuals. It appeared that 30 studies used chest X-ray images, 20 used chest CT images, one used chest X-ray and CT images, and one used chest ultrasound (US) frames. Thirteen of the included studies used ML models to predict COVID-19 disease severity [39,40,46,51,56,59,63,77,86,87,89,92]. Severity outcome measures included the need for ICU transfer, hospital stay time, mechanical ventilation, and death (Appendix 1).

Types of Machine Learning Techniques Used

Different ML methods were used in the studies. These included convolutional neural networks (CNNs), decision trees (DT), random forest (RF), gradient boosting machines (GBM), support vector machines (SVM), artificial neural networks (ANN), k-nearest neighbors (KNN), logistic regression, and naive Bayes (Appendix 1). Most studies used (DL) models (74%), specifically in the form of CNN. However, ML is not limited to this technique. Some studies used a combination of types of ML models to enhance the CNN model. In contrast, others compared the performance of different ML models to identify the one with superior diagnostic accuracy. Modifying pre-trained models was also popular among the retrieved studies. For the most part, model architecture was clearly described, and the breakdown of datasets to testing, training, and validation was also mentioned. This later information was missing from 15 studies.

Diagnostic Accuracy Measures

The reported measures of model accuracy varied across the retrieved studies (Appendix 1). These measures included accuracy, sensitivity, specificity, precision, recall, F1 score, positive predictive value (PPV), negative predictive value (NPV), the area under the curve (AUC), Kappa statistic, % correctness, and % completeness. The most frequently reported measure was accuracy. The majority of studies reported performance measures exceeding 90%. This was commonly reported in studies that utilized ML to diagnose COVID-19 through chest imaging. In all instances, ML accuracy was superior to the resident or consultant's naked-eye diagnosis.

Risk of Bias Assessment

**Figure 3:**
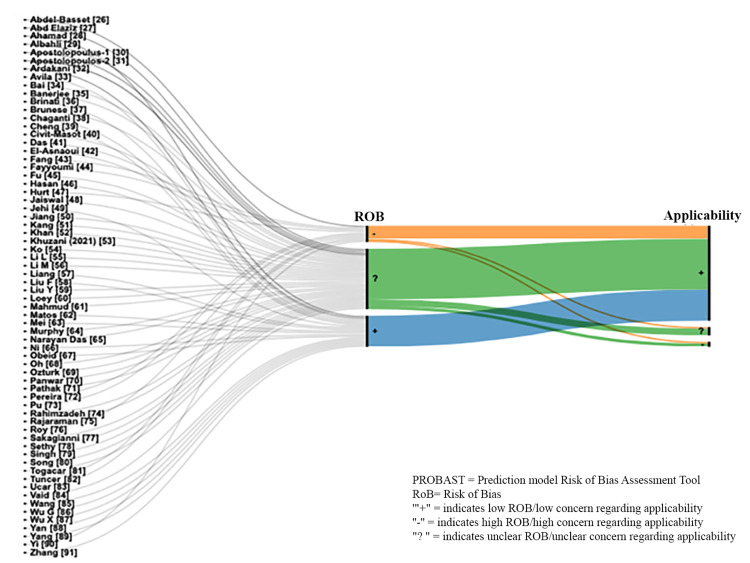
Critical appraisal of the selected studies based on the PROBAST PROBAST: Prediction model Risk Of Bias ASsessment Tool.

The overall risk of bias was "unclear" for 56% of the studies, while the applicability concerns were "low" for 88% of the studies (Figure 3).

Unfortunately, most of the studies fell short in the domains of participant selection. This was because the majority of studies, and more specifically those using secondary open-source data, selected their databases arbitrarily, and inclusion and exclusion criteria for the selected sample were never mentioned. Additionally, it was unclear why the databases were fixed or whether or not the subsamples (COVID-19 vs. no COVID-19) were randomly selected, which may have introduced selection bias to the studies. When data from different countries were used, it was unclear how comparable these data were. The predictor data were mostly chest images, and participants' characteristics were rarely considered during analysis. As most studies involved chest imaging, the timing of chest image acquisition was seldom recorded. The quality of chest image datasets was also questionable. For example, the images from the two most commonly used data sources (Joseph Cohen and Kaggle.com) are stored in JPEG format, which is of low 8-bit depth (256 gray shades), making them vulnerable to losing important pixel information. This imaging quality does not reflect the clinical or radiology practice where digital imaging and communications in medicine (DICOM) images of at least 12 bits (4096 gray shade) are used.

Most of the data used in these studies were from China, so it is not easy to assume similar accuracy if the ML models are tested on outside populations. On the other hand, other studies created large datasets, including data collected from different hospitals worldwide. Moreover, most studies did not specify the covariates controlled for in the ML models. This may have introduced confounding by unmeasured personal characteristics. The points above may have introduced bias to the evaluated studies' internal and external validity.

Discussion

Incorporating ML into health care is becoming more common. Advancements in ML have accelerated exceptionally during the COVID-19 pandemic, in which the technology has been adopted and improved for COVID-19 screening, diagnosis, and treatment, in addition to vaccine development [97,98]. The current systematic review focused on summarizing the published literature on utilizing ML in the diagnosis or prognosis of COVID-19 during the early phase of the pandemic. The findings from this study can be summarized in the following points. First, the studies suggest that ML can indeed help in identifying COVID-19 with high levels of accuracy, especially in the context of radiological diagnosis. Second, DL is the most preferred ML method for this purpose. Third, secondary data analysis was common among these studies as many researchers shared these data through open platforms. However, despite this data compilation, most of the data were collected from Chinese populations. There was little effort to merge large datasets to conduct ML testing on large samples representing various populations worldwide. The popularity of the application of ML on chest X-rays and CT scans in the retrieved studies agrees with what has been previously published [14,15,97,98]. This may be due to the feasibility of obtaining chest images in most healthcare settings. It may also be linked to the availability of open-source chest image data for training and the numerous existing pre-trained models that can be applied to chest images [14,15,97,98].

The literature examining the utility of ML in chest imaging suggests that this technology is exceptionally efficient in augmenting physicians' diagnoses, which can help reduce medical errors and improve patient safety [97,98]. Although abundant literature explores using ML to identify pulmonary lesions on chest imaging, there is still room for innovation in this domain. Future research could combine all available data from different countries into one mega-dataset and validate and test existing models for diagnostic accuracy. Another venue worth exploring would be pushing the accuracy of ML in identifying COVID-19 lesions using US chest imaging. This method is less invasive than conventional radiological approaches and has not been thoroughly examined in the currently reviewed literature [99]. DL is the most common ML method utilized as a decision support system for medical purposes [19,97,98]. DL has been described as having a shorter testing time when compared to other types of ML models. Additionally, many pre-trained DL models, particularly CNN models, have been shared during the pandemic as open-source algorithms that may have made it easier for other researchers to use as backbones to build on [98].

Given that most of the literature examined DL models using chest images as the main predictor, we recommend that future research expand on existing models and experiments with DL using presenting symptoms and laboratory markers as predictors. The retrieved studies suggest that the latter two indicators were used primarily in regression rather than DL modeling. The availability of COVID-19 data repositories may have driven the frequency of using secondary data for ML modeling. The urgency of expediting and facilitating COVID-19-related research during the early stages of the pandemic made scientific journals encourage authors to share their data through publicly accessible COVID-19 data repositories. Most of these include Chinese data, followed by data from the United States, the United Kingdom, and the European Union [100]. This may explain the abundance of Chinese data in the studies retrieved for our systematic review.

However, many publicly available chest X-ray data are stored in non-standard format with limited gray shade levels. This factor may limit the generalization of the used model. Chinese scientists also had the highest rate of COVID-19-related research production, especially in the early stages of the pandemic [100]. This may be explained by the natural course of the COVID-19 pandemic, which spread from China to other parts of the world two to three months later. Shuja et al. evaluated the sharing of COVID-19 datasets during the pandemic and identified 23 medical datasets shared for COVID-19 research [101]. Some of the mentioned drawbacks of these data included limited generalizability to other populations, small sample sizes, and challenges in accessing non-open-source data [101].

There are a few significant limitations to our study that should be mentioned. Due to the variability in ML models, datasets used, and accuracy measures, we could not synthesize pooled accuracy estimates. This variability also made it challenging to select the best method for ML modeling for prediction; different ML methods should be used depending on the context and desired prediction functions [98]. Moreover, our systematic review was limited to the search engines mentioned. Therefore, our review could have missed studies indexed outside these databases and in languages other than English. However, despite these limitations, this systematic review provided a detailed summary of the data types, predictive measures, and accuracy measures reported in ML models used to predict the diagnosis and prognosis of COVID-19 in the early pandemic phase. It also provided a detailed critique of the quality of the published literature, something lacking in many of the available reviews posted on this topic. We believe that our results can be used as a data source for future researchers to select existing models and publicly available data to experiment with in order to modify ML methods for enhancing healthcare delivery, especially with the new development in AI-Chatbots, such as ChatGPT, that was used to trigger possible causes of excess mortality in 2022 [102,103]. Further research is warranted on whether evolving AI-Chatbots could facilitate early integration of AI into future infectious disease outbreaks, provided these models become more reliable [104-106].

## Conclusions

The COVID-19 pandemic has caused unprecedented disruption to healthcare systems around the world. This has led many countries to adopt modern technological approaches that can be alternatives to high-cost and inaccessible medical investigations and management modalities for combating COVID-19. The research suggests that ML can serve as a helpful aid in localizing and segmenting COVID-19 lesions on chest images. However, due to the uncertainty around the selection of samples in such research and the ambiguity in controlling for essential confounders in the development of such ML models, the results of accuracy in disease prediction should be approached with caution. Nevertheless, this research is rapidly evolving and requires more efforts to validate and test the existing models to establish their efficacy in different population settings. Although this current technology should not replace the gold standard diagnostic method for COVID-19 via RT-PCR, we encourage researchers to continue the scientific battle against this pandemic, focusing their interests on developing large datasets from different countries on which the existing models can be tested. These can be formed into mega-data repositories. Finally, transparency about data sources and sampling techniques is also essential for scientists to improve the quality of ML diagnostic and prognostic research.

## Appendices

**Table 2:** Summary of published literature involving the diagnosis or prognosis of COVID-19 from January 2020 to June 2020 Dataset breakdown = Training, validation, and testing. N/A = Not available.

Study	Country	Study design	Study field	Study setting	Data type	Data source	Sample size	Dataset breakdown	Reported study aim/purpose	Diagnostic outcome of interest	Prognosis outcome of interest	Predictors of interest	Model development	Type of ML model	Reported tests for accuracy
Dabbagh et al. [26]	Egypt	Unnested case-control study	Computer science	Computer lab	Secondary dataset	Joseph Cohen dataset (https://github.com/ieee8023/covid-chestxray-dataset)	Eight chest X-ray images	N/A	Using an improved marine predators algorithm for the detection of COVID-19 on chest X-ray images	Diagnosis of COVID-19	N/A	Chest X-ray images	New model based on existing backbone	ML algorithm; marine predators’ algorithm with ranking-based diversity reduction	Average peak signal to noise ratio = 30.9; Average signal to noise ratio = 25.4; Structured similarity index metric = 0.98
Elaziz et al. [27]	Egypt	Unnested case-control study	Radiology	Hospital-based	Secondary dataset	(1) Joseph Cohen dataset; (2) Images from 43 publications (https://github.com/ieee8023/covid-chestxray-dataset/blob/master/metadata.csv); (3) Kaggle.com; (4) Chowdhury et al., 2020 data (from Qatar University, University of Dhakar and Malaysia); and (5) SIRM	Total 3,451 X-ray images—Dataset-1: 216 COVID-19 and 1,675 without COVID-19; Dataset-2: 219 COVID-19 and 1,341 without COVID-19	N/A	To propose a method for COVID-19 chest X-ray image classification	Diagnosis of COVID-19	N/A	Chest X-ray images	Developed new model	ML; DL; CNN A modified Manta-Ray Foraging Optimization (MRFO) based on differential evolution (DE) as a feature selection method.	Reported tests by dataset: Dataset-1 Accuracy = 96.09%; Recall = 98.75%; Precision = 98.75%; Dataset-2 Accuracy = 98.09%; Recall = 98.91%; Precision = 98.91%
Ahamad et al. [29]	China	Unnested case-control study	Internal medicine	Computer lab	Secondary dataset	BDBC-KG-NLP/COVID-19-tracker	Total 6,512 patients: 1,572 COVID-19 cases and 4,940 suspected cases	70% training; 30% testing	To predict COVID-19-positive patients among suspected and confirmed individuals	Diagnosis of COVID-19	N/A	Fever, cough, runny nose	Used existing pretrained models	ML; (1) Deep learning (DL); Extreme Gradient Boosting (XGBoost); (2) Decision Tree; (3) Random Forest; (4) Gradient Boosting Machine (GBM); (5) Support Vector Machine (SVM).	Accuracy for each model: XGBoost = 88%; Decision Tree = 6%; Random Forest = 86%; GBM = 86%; SVM = 86%
Albahli [30]	Saudi Arabia	Unnested case-control study	Radiology	Computer lab	Secondary dataset	(1) Joseph Cohen dataset; (2) Kaggle.com; (3) Synthetic dataset generated by GAN model	Total 119,968 chest X-ray images: 337 COVID-19, 1,026 pneumonia, 34,293 other lung conditions (atelectasis, effusion, infiltration, mass, nodule, pneumothorax, cardiomegaly), and 84,312 normal	N/A	To distinguish COVID-19 images from other chest diseases; to create a machine learning model with multiclass classification using X-rays	Diagnosis of COVID-19 and other pneumonia classification	N/A	Chest X-rays images	Updated existing models	ML; DL; Model 1: DL (CNN) with 4-layered convolutions for image augmentation; Model 2: Transfer learning model using IncpetionV3; Model 3: DL using ResNet without image augmentation; Model 4: DL with image augmentation and 8 targeted classes	Accuracy for each model: Model 1: Training = 65.67%; Validation = 66.11%. Model 2: Training = 65.51%; Validation = 66.43%. Model 3: Training = 89%; Validation = 59.81%. Model 4: Training = 81%; Validation = 89.2%.
Apostolopoulos et al. [31]	Greece	Unnested case-control study	Radiology	Computer lab	Secondary dataset	(1) Joseph Cohen dataset; (2) the Radiology Society of North America (RSNA); (3) Radiopaedia; (4) Italian Society of Medical and Interventional Radiology (SIRM); (5) Kermany et al. 2018 dataset; (6) National Institutes of Health X-ray data	Total 2,555 chest X-ray images: 455 COVID-19, 910 bacterial and viral pneumonia, and 1,190 pulmonary diseases (pleural effusion, emphysema, and COPD)	N/A	To extract features of lung disease from X-rays, including COVID-19	Diagnosis of COVID-19 and classification vs. pneumonia and other lung diseases	N/A	Chest X-ray images	Used existing pretrained models	ML; DL; CNN (MobileNet)	Sensitivity: 97.4%; Specificity: 99.4%. 2-class accuracy: 99.2%; 7-class accuracy = 87.7%
Apostolopoulos and Mpesiana [32]	Greece	Unnested case-control study	Radiology	Computer lab	Secondary dataset	(1) Joseph Cohen dataset; (2) RSNA; (3) Radiopaedia; (4) SIRM; (5) Kermany et al. 2018 dataset	Total 2869 chest X-ray images—Dataset-1: 224 COVID-19, 700 bacterial pneumonia, 504 normal; Dataset-2: 224 COVID-19, 417 bacterial and viral pneumonia, and 504 normal	N/A	To automatically diagnose COVID-19 from chest X-rays	Diagnosis of COVID-19 and classification vs. other pneumonia	N/A	Chest X-ray images	New model based on existing backbones	ML; DL; CNN (1) VGG19; (2) MobileNet-v2; (3) Inception; (4) Xception; (5) Inception-ResNet-v2	Performance measures for best performing model (MobileNet v2): Dataset 1: Sensitivity = 99.1%; Specificity = 97.1%; 2-class accuracy = 97.4%; 3- class accuracy = 92.9%; Dataset 2: Sensitivity = 98.7%; Specificity = 96.5%; 2-class accuracy = 96.8%; 3-class accuracy = 94.7%
Ardakani et al. [33]	Iran	Retrospective cohort	Radiology	Hospital-based	Primary dataset	Iran University Hospital	Total 194 patients: 108 COVID-19 cases, 86 pneumonia (Total 1020 chest CT images)	89% training (816; 50%-50% distribution), 11% validation (102; 50%-50% distribution)	To classify COVID-19 pneumonia vs. other viral or atypical pneumonia	Diagnosis COVID-19 and other pneumonia classification	N/A	Chest CT images	Used existing pretrained models	ML; DL; pre-trained convolutional neural network (CNN); (1) AlexNet; (2) VGG-16; (3) VGG-19; (4) SqueezeNet; (5) GoogleNet; (6) MobileNet-V2; (7) ResNet-18; (8) ResNet-50; (9) ResNet-101; (10) Xception	Performance measures for the best two CNN validation models: ResNet-101: Sensitivity = 100%; Specificity = 99.0%; Accuracy = 99.5%; PPV = 99.0%; NPV = 100%; AUC = 0.99. Xeption: Sensitivity = 98.0%; Specificity = 100%; Accuracy = 99.0%; PPV = 100%; NPV = 98.1%; AUC = 0.99.
Avila et al. [34]	Brazil	Retrospective cohort	Internal medicine	Hospital-based	Primary dataset	Hospital Israelita Albert Einstein (HIAE, Sao Paulo, Brazil)	Total 510 patients: 73 COVID-19 cases, 438 without COVID-19	N/A	To predict COVID-19 diagnosis using hemogram data	Diagnosis of COVID-19	N/A	Hematocrit, hemoglobin, platelets, eosinophils, neutrophils, basophils, lymphocytes, leukocytes, monocytes, red blood cell count (RBC), MCV, MCH, MCHC, MPV, RDW	New model based on existing backbone	ML; Bayes (Naive Bayes)	Best model performance when the prior probability was set to 0.2933; Sensitivity = 76.6%; Specificity = 76.6%; Accuracy = 76.6%; F1 score = 76.6%; AUC = 0.84
Bai et al. [35]	China	Unnested case-control study	Radiology	Hospital-based	Primary dataset	(1) Nine hospitals in Hunan Province, China; (2) Xiangya Hospital; (3) Rhode Island Hospital; (4) Hospital of the University of Pennsylvania	Total 1186 chest CT images: 521 COVID-19, 665 other pneumonia	70% training (830), 20% validation (237), and 10% testing (119)	To discriminate COVID-19 from other pneumonia on chest CT images	Diagnosis of COVID-19 and other pneumonia classification	N/A	Age, sex, temperature, white blood cell count (WBC), lymphocyte count, pre-existing conditions, duration of symptoms, source of transmission, COVID-19 severity, chest CT image	Used pretrained model	ML; DL; CNN EfficientNet B4 (a CNN pretrained on ImageNet)	Sensitivity = 95% (95% CI: 83%, 100%); Specificity = 96% (95% CI: 88%, 99%); Accuracy = 96% (95% CI:90%, 98%); AUC = 0.95
Banerjee et al. [36]	Brazil	Unnested case-control study	Internal medicine	Computer lab	Secondary dataset	Hospital Israelita Albert Einstein mindstream-AI challenge data	Total 598 patients: 81 COVID-19 cases, 517 COVID-19 negative, 188 other pneumonia	N/A	To predict COVID-19 diagnosis solely based on blood laboratory panel	Diagnosis of COVID-19	N/A	Age, hematocrit, hemoglobin, mean platelet volume (MPV), RBC, lymphocytes, leukocytes, basophils, neutrophils, monocytes, mean corpuscular hemoglobin (MCH), mean corpuscular hemoglobin concentration (MCHC), eosinophils, mean corpuscular volume (MCV), RBC distribution width	Used existing pretrained models	ML; (1) Random Forest; (2) Lassoelastic net regulized generalized (gmlnt) linear models; (3) DL; Artificial neural network (ANN)	Performance measures for predicting COVID-19 among admitted patients: Random forest: Sensitivity = 0.85; Specificity = 0.90; Accuracy = 0.91; AUC = 0.94. glmnet: Sensitivity = 0.92; Specificity = 0.93; Accuracy = 0.91; AUC = 0.94. ANN: Sensitivity = 0.85; Specificity = 0.94; Accuracy = 0.90; AUC = 0.95
Brinati et al. [37]	Italy	Retrospective cohort	Internal medicine	Hospital-based	Secondary dataset	The Scientific Institute for Research, Hospitalization and Healthcare (IRCCS)	Total 279 patients: 177 COVID-19 cases, 102 non-COVID patients	80% training, 20% testing/validation	To predict COVID-19 diagnosis using routine blood tests	Diagnosis of COVID-19	N/A	Age, gender, leukocytes, platelets, C-reactive protein, aspartate transaminase (AST), alanine aminotransferase (ALT), gamma-glutamyl transferase (GGT), lactate dehydrogenase (LDH), neutrophils, lymphocytes, monocytes, eosinophils, basophils	Used existing pretrained models	ML; (1) Decision tree (extremely randomized trees); (2) Instance-based, k-nearest neighbors (KNN); (3) logistic regression; (4) Naive Bayes; (5) Random forest; (6) SVM	Best performance for random forest model without controlling for gender: Sensitivity = 92%; Specificity = 65%; PV = 83%; Accuracy = 82%; AUC = 0.84
Brunese et al. [38]	China, Italy, Australia, and the USA	Unnested case-control study	Radiology	Hospital-based	Secondary dataset	(1) Joseph Cohen dataset; (2) Ozturk et al., 2020; (3) Wang et al. chest X-ray 8 dataset	Total 2773 chest X-ray images: 250 COVID-19, 2753 other pulmonary diseases	37% training (100 COVID, 1000 other pulmonary diseases), 37% testing (100 COVID, 1000 other pulmonary diseases), and 26% validation (50 COVID, 753 other pulmonary diseases)	To discriminate between generic pulmonary diseases and COVID-19 and to highlight the areas in the chest X-ray symptomatic of the COVID-19 disease	Diagnosis of COVID-19 and other pneumonia classification	N/A	Chest X-ray images	New model based on existing backbone	ML; DL; CNN Based on VGG-16 (i.e., Visual Geometry Group)	Sensitivity = 87%; Specificity = 94%; Accuracy = 98%; F1 score = 89%
Chaganti et al. [39]	USA	Unnested case-control study	Radiology	Hospital-based	Primary dataset and secondary dataset	(1) Multiple centers in USA, Canada, and Europe; (2) The National Lung Screening Trial; (3) the COPDGene study	Total 9749 chest CT images: 431 COVID-19, 174 pneumonia, 274 other interstitial lung disease	Training datasets were not mutually exclusive: testing (200), abnormality segmentation training (901), lung segmentation training (9223)	To automatically quantify chest CT abnormalities most often seen in COVID-19	N/A	Severity	Severity extent (lung severity score, opacity percentage)	Used existing pretrained models	ML; DL; CNN Deep image-to-image network (for lung segmentation); DenseUNet (for abnormality segmentation); multiple linear regression (for prediction)	No measures for test accuracy were reported
Cheng et al. [40]	USA	Retrospective cohort	Internal medicine	Hospital-based	Primary dataset	Mount Sinai Health System	Total 1,987 COVID-19 patients	70% training, 30% testing (equally balanced ICU and non-ICU patients)	To predict the risk of transfer of COVID-19 to the ICU within 24 hours	N/A	Transfer to ICU within 24 h of prediction	Periodic monitoring of vital signs RBC; serum biochemical tests; coagulation profile; ECG results	Used pretrained model	ML; ensemble; random forest model-derived class probabilities	Sensitivity = 72.8% (95% CI: 63.2, 81.1); Specificity = 76.3% (95% CI: 74.7, 77.9); Accuracy = 76.2% (95% CI: 74.6, 77.7); Precision = 10.5% (95% CI: 8.3, 12.9); NPV = 98.7% (95% CI: 98.1, 99.1); AUC = 0.80 (95% CI: 0.75, 0.84)
Civit-Masot et al. [41]	Spain	Unnested case-control study	Radiology	Computer lab	Secondary dataset	https://public.roboflow.ai/classification/ covid-19-and-pneumonia-scans	Total 396 chest X-ray images: 132 COVID-19, 132 other pneumonia, 132 normal	80% training; 316 (105 COVID; 105 normal; 106 pneumonia); 20% testing/validation; 80 (27 COVID; 27 normal; 26 pneumonia)	Identification of pneumonia and COVID-19 from chest X-ray images	Diagnosis of COVID-19 and other pneumonia classification	N/A	Chest X-ray images	New model based on existing backbone	ML; DL; CNN based on VGG-16 model using TensorFlow with Keras	Sensitivity = 86%; Specificity = 92%; Accuracy = 85%; Precision = 85%; F1 score = 85%
Das et al. [43]	India	Unnested case-control study	Radiology	Computer lab	Secondary dataset	(1) Joseph Cohen dataset; (2) Kaggle.com; (3) NIH TB CXR data	Total 6,845 chest X-rays: 162 COVID-19, 4280 bacterial and viral pneumonia, 342 TB from China, 58 TB from USA, 340 healthy from China, 80 healthy from USA, 1583 healthy from world	All data were divided into 10 subsamples. Training was applied on 9 of the subsamples (10% each). Testing was applied on the 10th subsample (10%).	To detect COVID-19 infection in chest X-rays and classify COVID-19 compared to other pneumonia or normal chest X-rays	Diagnosis of COVID-19	N/A	Chest X-ray images	New model based on existing backbone	ML; DL; proposed CNN model compared to pretrained models: Inception Net V3, ResNet50 and SVM, COVID-Net, Truncated inception net (study model)	Performance of study model classifying COVID-19 compared to normal, TB, and other pneumonia: Sensitivity = 88%; Specificity = 100%; Precision = 100%; F1 score = 93%; AUC = 0.94
El Asnaoui and Chawki [44]	Morocco	Unnested case-control study	Radiology	Computer lab	Secondary dataset	(1) Joseph Cohen dataset; (2) Kermany et al. 2018	Total 6,087 chest images (X-rays and CTs): 231 COVID-19, 1,493 viral pneumonia, 2,780 bacterial pneumonia, 1,583 normal	80% training, 20% validation	To assess the accuracy of deep learning in the early detection of COVID-19	Diagnosis of COVID-19	N/A	Chest X-rays and CT images	Used existing pretrained models	ML; DL; CNN multiple transfer learning models: Incpetion_Resnet_V2; DensNet201; Resnest50; Inception_V3; Mobilenet_V2 VGG16; VGG19	Highest performance was reported for Incpetion_Resnet_V2: Sensitivity = 92.11%; Specificity = 96.06%; Accuracy = 92.18%; Precision = 92.38%; F1 Score = 92.07%
Fang et al. [45]	China	Unnested case-control study	Radiology	Hospital-based	Primary dataset	Beijing Youan Hospital	Total 75 chest CT images: 49 COVID, 29 other pneumonia	67% training, 33% testing	Distinguishing COVID-19 pneumonia from other pneumonia	Diagnosis of COVID-19	N/A	Chest CT images	Developed new model	ML; SVM	AUC of the training set = 0.86; AUC of the testing set = 0.83
Fayyoumi et al. [46]	Jordan	Cross-sectional	Internal medicine	General population	Primary dataset	Online survey	Total 105 participants: 41 COVID-19, 64 non-COVID	N/A	To establish a reliable trusted model to predict the potential patients of COVID-19 by using either statistical or machine learning models	Diagnosis of COVID-19	N/A	Age, smoker (yes vs. no), positive chest X-ray, fever, sore throat, aches and pain, dry cough, nasal congestion, absence of smell, diarrhea or vomiting, breathing difficulty	Developed new model	ML; (1) Logistic regression; (2) SVM; (3) DL; CNN (multi-layer perception)	Performance measures for each model: (1) Logistic regression: Sensitivity = 100%; Specificity = 78.6%; Accuracy = 85%; Precision = 66.7%; G-mean = 88.6; (2) SVM Sensitivity = 91.7%; Specificity = 87.5%; Accuracy = 90%; Precision = 91.7%; G-mean = 89.5; (3) Multi-layer perception Sensitivity = 87.8%; Specificity = 93.7%; Accuracy = 91.6%; Precision = 90%; G-mean = 90.7
Fu et al. [47]	China	Retrospective cohort	Radiology	Hospital-based	Primary dataset	Hospital data source, N/A	Total 64 patients: 21 stable COVID-19 cases, 43 progressive COVID-19 cases, 6 COVID-19 negative	63 patients for training and 1 patient for testing	To quantify COVID-19 disease severity and predict disease progression trends	N/A	Stable vs. progressive patients (progression was not clearly defined)	Chest CT images	Developed new model	ML; K(K-1)/2 binary SVM	Sensitivity = 80.95%; Specificity = 74.42%; AUC = 0.83
Hasan et al. [48]	Iraq	Unnested case-control study	Radiology	Computer lab	Secondary dataset	(1) Radiopaedia; (2) the cancer imaging archive (TCIA) websites	Total 321 chest CT images: 118 COVID-19, 96 other pneumonia, 107 normal	70% training, 30% validation/testing	To reduce the erroneous diagnostic interpretation of CT lung scans and assist clinicians to quickly discriminate patients who have COVID-19 from healthy ones	Diagnosis COVID-19 vs. other pneumonia	N/A	Chest CT images	Developed new model	ML; DL; For extraction: CNN in combination with Q-deformed entropy feature extraction (QDE). For classification: Long short-term memory, CNN classifier	Performance measures for classification model: Sensitivity = 100%; Accuracy = 99.7%
Hurt et al. [49]	USA	Case-series	Radiology	Computer lab	Secondary dataset	Five US and Chinese epidemiologic and case-study publications	Total of 10 chest X-rays from 5 patients	N/A	Assessing the generalizability of a DL algorithm on frontal chest X-ray images to diagnose pneumonia	Diagnosis of COVID-19 vs. other pneumonia	N/A	Chest X-ray images	Used existing pretrained model	ML; DL; CNN (U-Net)	Not reported
Jaiswal et al. [50]	Brazil	Unnested case-control study	Radiology	Computer lab	Secondary dataset	Kaggle.com	Total 2492 CT chest images: 1262 COVID-19, 1230 normal	68% training, 17% validation, 15% testing	To classify COVID-19 pneumonia vs. non-COVID-19 on chest CT images	Diagnosis of COVID-19	N/A	Chest CT images	New model based on existing backbone	ML; DL; CNN based on DenseNet201 architecture (the proposed model was compared to VGG16, ResNet5272 and Inception-ResNetV2)	Performance measures for testing model of DenseNet201: Specificity = 96.2%; Accuracy = 96.3%; Precision = 96.3%; F1 score = 96.3%; Recall = 96.3%; AUC = 0.97
Jehi et al. [51]	USA	Retrospective cohort	Internal medicine	Hospital-based	Secondary dataset	Cleveland Clinic COVID-19 registry	Total 11672 patients: 1108 COVID-19, 12,859 non-COVID	83.6% training, 11,672 (818 COVID-19; 10,854 non-COVID); 16.4% validation/testing, 2,295 (290 COVID-19; 2005 non-COVID)	To predict the individualized risk for testing positive for COVID-19	Diagnosis of COVID-19	N/A	Demographic characteristics, comorbidities, immunization history, symptoms, travel history, laboratory variables, medications	New model based on existing backbone	ML; Logistic regression	Sensitivity = 76.2%; Specificity = 76.5; PPV = 31.9%; NPV = 95.7%
Jiang et al. [52]	China	Case-series	Internal medicine	Hospital-based	Primary dataset	(1) Wenzhou Central Hospital; (2) Cangnan People's Hospital	Total 53 COVID-19 patients	N/A	(1) To identify the combinations of clinical characteristics of COVID-19 that predict outcomes; (2) to predict patients at risk for more severe illness on initial presentation	N/A	Acute respiratory distress syndrome (ARDS)	Alanine aminotransferase, myalgias, hemoglobin, gender, temperature, sodium, potassium, lymphocyte count, creatinine, age, WBC	Used existing pretrained models	ML; (1) Logistic regression; (2) KNN; (3) Decision tree (based on gain ratio); (4) Decision tree (based on Gini index); (5) Random forests; (6) SVM	Accuracy for each measure: Logistic regression model = 50%; KNN = 80%; Decision tree based on gain ratio = 70%; Decision tree based on gini index = 70%; Random forests = 70%; SVM = 80%
Kang et al. [53]	China	Unnested case-control study	Radiology	Hospital-based	Primary dataset	(1) Tongji Hospital of Huazhong University of Science and Technology; (2) China-Japan Union Hospital of Jilin University; (3) Ruijin Hospital of Shanghai Jiao Tong University	Total 2,522 chest CT images: 1,495 COVID-19, 1,027 community-acquired pneumonia	70% training, 30% testing	Diagnosis of COVID-19 pneumonia vs. community-acquired pneumonia on CT chest images	Diagnosis of COVID-19	N/A	Chest CT images	Updated existing models	ML; DL; Latent-representation-based CNN developed from CPM-Nets architecture (model was compared to logistic regression, SVM, Gaussian-Naive-Bayes, and KNN)	Performance of the proposed model: Sensitivity = 96.6%; Specificity = 93.2%; Accuracy = 95.5%
Khan et al. [54]	India	Unnested case-control study	Radiology	Computer lab	Secondary dataset	(1) Joseph Cohen dataset; (2) Kaggle.com; (3) Ozturk et al. 2020	Total 2128 chest X-ray images: 441 COVID-19, 1157 other pneumonia, 810 normal	46% training (284 COVID-19, 657 other pneumonia, 310 normal); 54% testing (157 COVID-19, 500 other pneumonia, 500 normal)	To classify COVID-19 pneumonia vs. other types of pneumonia	Diagnosis of COVID-19	N/A	Chest X-ray images	New model based on existing backbone	ML; DL; CoroNet (a CNN model based on Xception architecture)	Average performance of CoroNet on testing dataset: Specificity = 95.3%; Accuracy = 90.2%; Precision = 92%; F1 score = 91%; Recall = 90%
Khuzani et al. [55]	USA	Unnested case-control study	Radiology	Computer lab	Secondary dataset	(1) Joseph Cohen dataset; (2) Kermany et al. 2018 dataset	Total 140 chest X-ray images for COVID-19 patients	80% training, 20% testing	To develop a COVID-19 chest X-ray classifier to be implemented as an adjunct to other tests to facilitate differential diagnosis of chest X-ray images of COVID-19	Diagnosis of COVID-19 and other pneumonia classification	N/A	Chest X-ray images	Developed new model	ML; DL; multilayer CNN model	Sensitivity = 100%; Precision = 96%; F1 score = 98%
Ko et al. [56]	Republic of Korea and Italy	Unnested case-control study	Radiology	Hospital-based	Primary dataset and secondary dataset	(1) Wonkwang University Hospital; (2) Chonnam National University Hospital; (3) SIRM	Total 4257 chest CT images: 1458 COVID-19, 1357 other pneumonia, 998 normal, 444 lung cancer	75% training (955 COVID-19, 1086 other pneumonia, 698 normal, 355 lung cancer); 25% testing (503 COVID-19, 271 other pneumonia, 200 normal, 89 lung cancer)	To diagnose COVID-19 pneumonia in chest CT images and differentiate it from non-COVID-19 pneumonia and non-pneumonia diseases	Diagnosis of COVID-19	N/A	Chest CT images	New model based on existing backbone	ML; DL; CNN fast-track COVID-10 classification network (FCONet); four proposed transfer learning models were developed based on the architecture of each of VGG16, ResNet-50, Inception-v3, and Xception	FCONet based on ResNet-50: Sensitivity = 99.6%; Specificity = 100%; Accuracy = 99.9%; AUC = 1.00. FCONet based on VGG16: Sensitivity = 100%; Specificity = 99.6%; Accuracy = 99.7%; AUC = 1.00. FCONet based on Xception: Sensitivity = 97.9%; Specificity = 99.3%; Accuracy = 98.9%; AUC = 0.99. FCONet based on Inception-v3: Sensitivity = 88.3%; Specificity = 97.9%; Accuracy = 94.9%; AUC = 0.97
Li et al. [57]	China	Unnested case-control study	Radiology	Hospital-based	Primary dataset	Six unspecified medical centers in China	Total 4,356 chest CT images: 1,296 COVID-19, 1,735 community-acquired pneumonia, 1,325 normal	90% training, 10% testing	To detect COVID-19 pneumonia on chest CT	Diagnosis of COVID-19	N/A	Chest CT images	New model based on existing backbone	ML; DL; CNN COVNet (a CNN model based on pretrained RestNet50 model as a backbone)	Sensitivity = 90% (95% CI:83%, 94%); Specificity = 96% (95% CI:93%, 98%); AUC = 0.95 (95% CI: 0.94, 0.99)
Li et al. [58]	USA	Unnested case-control study	Radiology	Hospital-based	Primary dataset and secondary dataset	(1) Massachusetts General Hospital (internal data); (2) Stanford Hospital (external data for training and validation)	Total 581 chest X-rays: 314 for training and validation (internal dataset); 154 for testing (internal dataset); 113 for testing (external dataset)	54% training/validation, 46% testing	To develop a pulmonary X-ray severity score that predicts the severity of pulmonary disease	N/A	Intubation death	Pulmonary X-ray, severity score	New model based on existing backbone	ML; DL; CNN Convolutional Siamese Neural Network (a CNN model based on DensNet121 underlying subnetwork with initial pretraining on ImageNet)	Internal dataset: Pearson's correlation coefficient = 0.86 (95% CI: 0.80-0.90); Spearman's rank correlation coefficient = 0.84 (95% CI: 0.77-0.88). External dataset: Pearson's correlation coefficient = 0.86 (95% CI: 0.79-0.90); Spearman's rank correlation coefficient = 0.78 (95% CI: 0.67-0.85)
Liang et al. [59]	China	Retrospective cohort	Internal medicine	Hospital-based	Primary dataset	National Health Commission (NHC) of the People's Republic of China	Total 3,089 COVID-19 cases: 1,590 from 31 China provinces (1,459 not critical, 131 critical); 1,034 from Wuhan (940 not critical, 94 critical); 389 from Hubei province excluding Wuhan (380 not critical, 9 critical); 76 from Guangdong province (73 not critical, 3 critical)	80% training, 20% validation	To predict clinical outcomes of COVID-19	N/A	Critical illness (definition was not clear)	Age, dyspnea, COPD, cancer history, number of comorbidities, X-ray abnormality, neutrophil-lymphocyte ratio, LDH, direct bilirubin, creatine kinase	Developed new model	ML; DL; deep learning survival cox model	Performance measures for validation: Wuhan data: AUC = 0.89 (95% CI: 0.86, 0.91); Concordance index = 89.0% (95% CI: 86%, 91%). Huebi data: AUC = 0.88 (95% CI: 0.73, 0.98); Concordance index = 85.2% (95% CI: 67%, 97%). Guangdong data: AUC = 0.96 (95% CI: 0.90-1.00); Concordance index = 96.7% (95% CI: 90%, 100%)
Liu et al. [60]	China	Retrospective cohort	Radiology and internal medicine	Hospital-based	Primary dataset	The Shanghai Public Health Center	Total 134 COVID-19 cases	N/A	To compare the capability of quantitative CT imaging to biological markers in the prediction of the progression of COVID-19	N/A	Any severe event based on one major criterion (e.g., respiratory failure requiring mechanical ventilation, shock needing vasopressors, or extracorporeal membrane oxygenation), two or more minor criteria (e.g., a respiratory rate greater than 30 breaths/min, or O2 saturation lower than 93%), or two criteria of additional organ damage	Age, gender, APACHE-II, neutrophil-lymphocyte ratio, D-dimer, CT features, NLR combined with CT features	Developed new model	ML; regression (1) logistic regression (LR); (2) Cox proportional hazard model	AUC for each predictor (LR model): APACHE-II: AUC = 0.82 (95% CI: 0.72, 0.91); NLR: AUC = 0.78 (95% CI: 0.67, 0.88); d-dimer: AUC = 0.78 (95% CI: 0.67, 0.88). CT features: AUC = 0.93 (95% CI: 0.87, 0.99). Concordance indices (C-index) for each predictor (Cox model): APACHE-II: C-index = 80% (95% CI: 70, 90); NLR: C-index = 76% (95% CI: 66, 85); d-dimer: C-index = 76% (95% CI: 66, 86); CT features: C-index = 0.88 (95% CI:0.81, 0.95)
Liu et al. [61]	China	Retrospective cohort	Internal medicine	Hospital-based	Primary dataset	The First Affiliated Hospital of Zhejiang University	Total of 2243 patients visiting the fever clinic: 17 confirmed COVID-19, 2226 COVID-19 negative	N/A	To develop a dynamic risk assessment decision support system for COVID-19 (DDC19) to assist GPs in data collection, dynamic risk assessment, triage management, and follow-up	Classification of patients into low-risk, moderate-risk, and high-risk for COVID-19	N/A	Demographic data, exposure history, symptoms, laboratory data, chest CT images	Developed new model	ML; (1) NLP (for data extraction from patient history); (2) multiclass logistic regression	Performance of the saturated regression model: (1) Prediction of low-risk: Precision = 94.9%; F1 score = 95.2%; Recall = 95.6%; (2) Prediction of moderate-risk: Precision = 98%; F1 score = 96.8%; Recall = 95.7%; (3) Prediction of high-risk: Precision = 85%; F1 score = 90.9%; Recall = 98.2%
Loey et al. [62]	Egypt	Unnested case-control study	Radiology	Hospital-based	Secondary dataset	Joseph Cohen dataset	Total 306 chest X-ray images: 69 COVID-19, 79 bacterial pneumonia, 79 viral pneumonia, 79 normal	88% training (60 COVID; 70 normal; 70 bacterial pneumonia; 70 viral pneumonia); 12% testing (9 COVID; 9 normal; 9 bacterial pneumonia; 9 viral pneumonia)	To classify COVID-19 pneumonia compared to normal lung, other viral pneumonia, or bacterial pneumonia	Diagnosis of COVID-19 vs. other pneumonia	N/A	Chest X-ray images	Used existing pretrained models	ML; DL; three deep learning transfer CNN models (Alexnet; Googlenet; Resnet18) augmented with generative adversarial network (GAN)	For all three models: Accuracy = 100%; Precision = 100%; Recall = 100%
Mahmud et al. [64]	Bangladesh	Unnested case-control study	Radiology	Computer lab	Secondary dataset	(1) Guangzhou Medical Center, China; (2) Sylhet Medical College, Bangladesh	Total 5,856 chest X-ray images: 305 COVID-19; 1,493 other viral pneumonia; 2,780 bacterial pneumonia; 1,583 normal	N/A	Detecting COVID-19 from chest X-ray images	Diagnosis of COVID-19	N/A	Chest X-ray images	Developed new model	ML; DL; A CNN named as CovXNet,	Four-way classification (COVID-19 vs. bacterial pneumonia, vs. viral pneumonia vs. normal): Accuracy = 90.2%; Specificity = 89.1%; Precision = 90.8%; AUC = 0.91; Recall = 89.9%; F1 score = 90.4%
Matos et al. [65]	Italy	Retrospective cohort	Radiology and internal medicine	Hospital-based	Primary dataset	Hospital not specified	Total 106 COVID-19 cases	75% training, 25% testing	Analyzing the performance of combining quantitative CT with clinical and laboratory data to predict COVID-19 adverse clinical outcomes	N/A	Adverse outcome (defined as the need for mechanical ventilation or death)	Age, gender, duration of symptoms, WBC, lymphocyte percentage, C-reactive protein, volume of disease (extracted using autoselect function and expressed in cubic cm), predominant opacity type on CT (ground-glass opacities or consolidation), chronic lung disease (emphysema or fibrosis), coronary calcification, aortic calcification, presence of chronic comorbidity	Developed new models	(1) ML; logistic regression (penalized binomial regression); (2) GLM 2-conditional inference trees; (3) support vector machine with linear kernel	Penalized binomial regression: Sensitivity = 70%; Specificity = 87%; Accuracy = 81%; AUC = 0.91; PPV = 78%; NPV = 82%; GLM: Sensitivity = 80%; Specificity = 87%; Accuracy = 85%; AUC = 0.90; PPV = 80%; NPV = 87%. Conditional inference trees: Sensitivity = 100%; Specificity = 56%; Accuracy = 73%; AUC = 0.89; PPV = 59%; NPV = 100%. Support vector machine with linear kernel: Sensitivity = 90%; Specificity = 87%; Accuracy = 88%; AUC = 0.92; PPV = 82%; NPV = 93%
Mei et al. [66]	USA	Retrospective cohort	Radiology and internal medicine	Hospital	Primary dataset	Eighteen medical centers in 13 provinces in China	Total 905 patients: 419 COVID-19 cases, 486 COVID-19 negative	60% training (242 COVID-19 cases, 292 COVID-19 negative), 10% tuning (43 COVID-19 cases, 49 COVID-19 negative), 30% testing (134 COVID-19 cases, 145 COVID-19 negative)	Detection of COVID-19 infection at an early stage using initial CT scan and clinical information	Diagnosis of COVID-19	N/A	Chest CT images	Developed new models	ML; DL; multilayer perceptron classifier (MLP) joining model: This consisted of a CNN model for identifying CT images combined with random forest and support vector machines for integrating it with clinical information for prediction of COVID-19	Sensitivity = 84.3% (95% CI: 77.1-90.0); Specificity = 82.8% (95% CI: 75.6-88.5); AUC = 0.92 (95% CI: 0.88-0.94)
Murphy et al. [67]	Netherlands	Unnested case-control study	Radiology	Hospital	Primary dataset and secondary dataset	(1) Kaggle.com (training and validation); (2) Bernhoven Hospital (training); (3) Radboud University Medical Center (training); (4) Jeroin Bosch Hospital (testing)	Total 25,146 chest X-ray images: 416 COVID-19 cases, 191 COVID-19 negative, 468 COVID-19 suspected cases, 7,851 normal, 5,012 pneumonia, 9,321 abnormal but inconsistent with pneumonia	92% training (23,138), 6% validation (1,540), 2% testing (468)	Detection of COVID-19 pneumonia on chest X-ray	Diagnosis of COVID-19	N/A	Chest X-ray images	Used existing pretrained model	ML; DL; CAD4COVID-X-ray	At 85% sensitivity: Specificity = 61%; PPV = 68%; NPV = 81%
Das et al. [68]	India	Unnested case-control study	Radiology	Computer lab	Secondary dataset	(1) Joseph Cohen dataset; (2) Chest X-ray8 data by Wang et al.	N/A	70% training, 10% validation, 20% testing	Develop an automated deep learning-based approach for the detection of infection in chest X-rays	Diagnosis of COVID-19	N/A	Chest X-ray images	New model based on existing backbone	ML; DL; deep transfer learning developed by combining CNN and Xeption model	Performance of testing of proposed model: Sensitivity = 96.1%; Specificity = 97.3%; Accuracy = 97.4%; F-measure = 0.97; Kappa statistic = 0.97
Ni et al. [69]	China	Unnested case-control study	Radiology	Hospital-based	Primary dataset and secondary dataset	(1) Training and validation data were retrieved from commercially available data from Yu et al. 2020; (2) Taihe Hospital, Shiyan, Hubei; (3) Wuhan First Hospital, Wuhan, Hubei; (4) Jinling Hospital, Nanjing, Jiangsu	Total 19,387 CT chest images: 3,950 COVID-19 pneumonia, 6,871 other pneumonia, 8,566 normal	99.5% training and validation (19,291), 0.5% testing (96)	Detect COVID-19 pneumonia lesions on chest CT	Diagnosis of COVID-19	N/A	Chest CT images	Used existing pretrained models	ML; DL; pre-trained CNN MVP-Net (for abnormality detection); 3D U-Net (for lobe segmentation)	Performance of model for abnormality detection: Sensitivity = 100% (95% CI: 96%, 100%); Specificity = 25% (95% CI: 3%, 65%); Accuracy = 94% (95% CI: 87%, 98%); PPV = 94% (95% CI: 91%, 96%); NPV = 100% (95% CI: 63%, 100%); F1 score = 97%
Obeid et al. [70]	USA	Retrospective cohort	Internal medicine	Virtual care	Primary dataset	Medical University of South Carolina Health System virtual care	Total 6,813 patients: 498 COVID-19 cases, 6,315 COVID-19 negative	60% training, 16% validation, 24% testing	To improve the COVID-19 screening process at virtual care visits, using deep learning model	Diagnosis of COVID-19	N/A	Text sequences from patient record notes	New model based on existing backbones	ML; (1) DL; CNN; (2) regression; logistic regression	CNN model: AUC = 73.2% (95% CI: 69.7%, 76.7%); Precision = 75.4%; Recall = 45.3%; F1 score = 56.6% (95% CI: 54.1%, 58.6%). Logistic regression model: AUC = 70.7% (95% CI: 66.5%, 73.9%); Precision = 80.0%; Recall = 45.3%; F1 score = 56.6% (95% CI: 54.1%, 58.6%)
Oh et al. [71]	Republic of Korea	Unnested case-control study	Radiology	Computer Lab	Secondary dataset	(1) Japanese Society of Radiological Technology; (2) US National Library of Medicine (Montgomery Country dataset); (3) Corona hack: chest X-ray dataset; (4) Joseph Cohen dataset	Total 502 chest X-ray images: 180 COVID-19, 20 other viral pneumonia, 57 tuberculosis, 54 other bacterial pneumonia, 19 normal	70% training (345), 10% validation (49), 20% testing (99)	To classify chest X-ray images according to disease types (COVID-19 pneumonia, other viral pneumonia, TB pneumonia, and other bacterial pneumonia)	Diagnosis of COVID-19	N/A	Chest X-ray images	Used existing pretrained model	ML; DL: CNN Pretrained ResNet-18 model	Specificity = 96.4%; Accuracy = 88.9%; Precision = 83.4%; Recall = 85.9%; F1 score = 84.4%
Ozturk et al. [72]	Turkey	Unnested case-control study	Radiology	Computer lab	Secondary dataset	(1) Joseph Cohen dataset; (2) ChestX-ray8 data by Wang et al. 2017	Total 1127 chest X-ray images: 127 COVID-19, 500 other pneumonia, 500 normal	80% training (902), 20% validation (225)	Automated diagnosis of COVID-19 from chest X-ray images	Diagnosis of COVID-19 and classification from other pneumonia classes	N/A	Chest X-ray images	New model based on existing backbone	ML; DL: CNN DarkCOVIIDNet, which is based on the Darknet-19 architecture	Sensitivity = 85.3%; Specificity = 92.2%; Precision = 89.9%; Accuracy = 87.0%; F1 score = 87.4%
Panwar et al. [73]	India	Unnested case-control study	Radiology	Computer lab	Secondary dataset	(1) Joseph Cohen dataset; (2) Kaggle.com (for normal images)	Total 284 chest X-ray images: 142 COVID-19, 142 normal	70% training, 30% testing	Detecting COVID-19 from chest X-ray images	Diagnosis of COVID-19	N/A	Chest X-ray images	New model based on existing backbone	ML; DL, CNN based on nCOVnet	Sensitivity = 97.6%; Specificity = 78.6%; PPV = 97.6%; AUC = 0.88
Pathak et al. [74]	India	Unnested case-control study	Radiology	Hospital-based	Secondary dataset	(1) Chowdhury et al. 2020; (2) Dilbag et al. 2020	Total 852 chest X-ray images: 413 COVID-19, 439 normal or pneumonia	60% training and validation (9:1 ratio), 40% testing	To classify COVID-19 compared to normal lung or pneumonia using CT images	Diagnosis of COVID-19	N/A	Chest CT images	Used existing pretrained model	ML; DL: deep transfer learning model (ResNet-50)	Sensitivity = 89.6%; Specificity = 92.0%; Accuracy = 90.8%; Precision = 92.6%; NPV = 88.8%
Pereira et al. [75]	Brazil	Unnested case-control study	Radiology	Computer lab	Secondary dataset	(1) Joseph Cohen dataset; (2) Radiopaedia encyclopedia; (3) NIH Chest X-ray14 dataset	Total 1144 chest X-ray images: 90 COVID-19, 10 MERS-CoV, 11 SARS-CoV, 10 Varicella, 12 Streptococcus, 11 pneumocystis	70% training, 30% testing	To identify pneumonia caused by COVID-19 from other types and also healthy lungs using only CXR images	Diagnosis of COVID-19 and other pneumonia classification	N/A	Chest X-ray images	Used existing pretrained models	ML; (1) multiclass classification: This used k-Nearest neighbors (kNN); Support Vectors Machine (SVM); Multilayer Perceptrons; Decision Trees and Random Forests. (2) Hierarchial classification: Clus-HMC, which is based on predictive cluster trees	For the multi-class classification: F1 score ranged from 80.0% to 83.3% depending on the fusion scenario. For hierarchical classification: F1 score ranged from 82.7% to 88.9% depending on the fusion scenario
Pu et al. [76]	China	Unnested case-control study	Radiology	Hospital-based	Primary dataset	Not specified	Total 649 chest CT images: 151 COVID-19, 498 not COVID-19	75% training (97 COVID-19; 393 not COVID-19), 13% validation (27 COVID-19; 55 not COVID-19), 12% testing (27 COVID-19; 50 not COVID-19)	To classify COVID-19 from community-acquired pneumonia using CT images	Diagnosis of COVID-19 and other pneumonia classification	N/A	Chest CT images	Developed new models	ML; DL: 3D CNN models	Sensitivity = 28% (8%-50%); Specificity = 98%; AUC = 70% (56%-85%)
Rahimzadeh et al. [77]	Iran	Unnested case-control study	Radiology	Computer lab	Secondary dataset	(1) Joseph Cohen dataset; (2) Kaggle.com	Total 15043 chest X-ray images: 180 COVID-19, 6012 pneumonia, 8851 normal	25% training (149 COVID-19, 1634 pneumonia, 2000 normal); 75% validation (31 COVID-19, 4420 pneumonia, 6851 normal)	To detect COVID-19 on chest X-ray images	Diagnosis of COVID-19	N/A	Chest X-ray images	New model based on existing backbones	ML; DL; CNN. The CNN used was developed by concatenating the extracted features of Xception and ResNet50V2	Average performance measures for detecting COVID-19 by the concatenated model: Sensitivity = 80.5%; Specificity = 99.6%; Accuracy = 99.5%; Precision = 35.3%
Rajaraman and Antani [78]	USA	Unnested case-control study	Radiology	Computer lab	Secondary dataset	(1) Kermany et al. 2018 (pediatric chest X-ray dataset); (2) NIH chest X-ray14 dataset; (3) CheXpert chest X-ray dataset (from Stanford Hospital, California); (4) Twitter COVID-19 chest X-ray dataset; (5) Montreal COVID-19 chest X-ray dataset	Total 15589 chest X-ray images: 314 COVID-19, 2780 pediatric bacterial pneumonia, 1493 pediatric viral pneumonia, 11002 adult pneumonia of unknown type	95.5% training (3883 pediatric data, 11002 adult pneumonia of unknown type), 4.5% testing (314 COVID-19, 390 pediatric data)	Classification of COVID-19 pneumonia as a viral pneumonia	Diagnosis of COVID-19	N/A	Chest X-ray images	New model based on existing backbone	DL; CNN: Custom wide residual network CNN model vs. the following: (1) VGG-16; (2) Inception-V3; (3) Xception; (4) DenseNet-121; (5) NasNet-mobile	Accuracy (Only reported for VGG-16 model): For twitter-COVID-19 data: Accuracy = 0.2885. For Montreal-COVID-19 data: Accuracy = 0.5028
Roy et al. [79]	Italy	Retrospective cohort	Radiology	Hospital setting	Secondary dataset	Italian COVID-19 Lung Ultrasound Database (ICLUS-DB)	Total of 35 patients: 17 confirmed COVID-19, 4 suspected COVID-19 14 without COVID-10	70% training, 30% testing	To use lung ultrasound to predict the presence or absence of pathological artifacts and assess the severity of COVID-19 disease according to COVID-19 lung ultrasound scoring system	Pathological scoring for COVID-19 pneumonia	Severity of COVID-19	Ultrasound frames	New models based on existing backbones	ML; DL; three DL models: (1) Frame-Based Score Prediction Evaluation model (formed of CNN combined with Regularized Spatial Networks and soft ordinal regression); (2) video-based score prediction evaluation model (soft ordinal regression); (3) semantic segmentation model (combination of U-net, U-net++, and Deeplab v3+)	Model 1: Fa score = 71.4%. Model 2: F1 score = 61%; Precision = 70%; Recall = 60%. Model 3: Accuracy = 96%; Binary Dice score = 0.75
Sakagianni et al. [80]	Greece	Unnested case-control study	Radiology	Computer lab	Secondary dataset	COVID-CT-Dataset	Total 746 chest CT images: 349 COVID-19, 397 without COVID-19	80% training (279 COVID-19 and 317 non-COVID), 10% validation (34 COVID-19 and 39 non-COVID), 10% testing (36 COVID-19 and 41 non-COVID)	To diagnose COVID-19 pneumonia using chest CT scans	Diagnosis of COVID-19	N/A	Chest CT images	Used existing pretrained model	ML; Google AutoML Cloud Vision	Sensitivity = 88.6%; Specificity = 88.1%; F1 score = 85%; AUC = 0.94
Sethy and Behera [81]	India	Unnested case-control study	Radiology	Computer lab	Secondary dataset	(1) Joseph Cohen dataset; (2) Kaggle.com; (3) Kermany et al. 2018	Total 381 chest X-ray images: 127 COVID-19, 127 pneumonia, 127 normal	80% training, 20% testing	To classify COVID-19 and pneumonia using chest X-ray images	Diagnosis of COVID-19 vs. other pneumonia classification	N/A	Chest X-ray images	Used existing pretrained models	ML; DL; the following pre-trained models were extracted using SVM: (1) AlexNet; (2) Vgg16; (3) Vgg19; (4) MobileNetV2; (5) ShuffleNet; (6) Xception; (7) Resnet18; (8) Resnet50; (9) Resnet101; (10) Inceptionv3; (11) Inceptionresnetv2; (12) GoogleNet; (13) Densnet201	Best performance measures were reported for Resnet50: Mean accuracy = 95.33%; Sensitivity = 95.33%; False positive rate = 2.33; F1 score = 95.34%
Singh et al. [82]	India	Unnested case-control study	Radiology	Computer lab	Secondary dataset	Not specified	Total 133 chest CT images: 68 COVID-19, 65 normal	Multiple were applied: 20% training; 80% testing, 30% training; 70% testing, 40% training; 60% testing, 50% training; 50% testing, 60% training; 40% testing, 70% training; 30% testing, 80% training; 20% testing, 90% training; 10% testing	To classify COVID-19 using chest CT images	Diagnosis of COVID-19	N/A	Chest CT images	Developed new model	ML; DL; CNN multi-objective differential evolution (MODE)–based CNN	The highest performance was reported for training to testing ratio of 90:10: Sensitivity = 90.8%; Specificity = 90.7%; Accuracy = 93.4%; F1 score = 98.88%; Kappa statistic = 90.5%
Song et al. [83]	China	Unnested case-control study	Radiology	Hospital	Primary dataset	(1) The first affiliated hospital to the University of Science and Technology of China; (2) The Lu'an affiliated hospital of Anhui Medical University in China	Total 201 patients: 98 COVID-19 pneumonia, 103 non-COVID pneumonia	80% training, 10% validation, 10% testing	To differentiate COVID-19 pneumonia from normal lung	Diagnosis of COVID-19 and other pneumonia classification	N/A	Chest CT images	Used existing pretrained models	ML; DL; (1) bi-directional generative adversarial network (BigBiGAN); (2) SVM; (3) KNN	Performance measures in testing dataset: (1) For BigBiGAN: AUC = 0.972; Sensitivity = 92%; Specificity = 91%. (2) For SVM model: AUC = 0.531. (3) KNN model: AUC = 0.998; Sensitivity and specificity of radiologists to diagnose COVID-19 when assisted by BigBiGAN increased by 8%, and 13%, respectively.
Toğaçar et al. [84]	Turkey	Unnested case-control study	Radiology	Computer lab	Secondary dataset	(1) Joseph Cohen dataset; (2) Kaggle.com	Total 428 chest X-ray images: 295 COVID-19, 68 non-COVID pneumonia, 65 normal	70% training, 30% testing; k-fold cross-validation was applied as the last step.	To differentiate COVID-19 pneumonia from other pneumonia and normal lung	Diagnosis of COVID-19 and other pneumonia classification	N/A	Chest X-ray images	Used existing pretrained models	ML; DL; CNN (1); MobileNet2 (2); SqueezeNet ML; SVM with stochastic gradient descent for classification	Reported performance measures for using the stacked data technique in combination with the proposed models: (1) For MobileNet2: Sensitivity = 98.8%; Specificity = 95.7%; Precision = 97.8%; Accuracy = 97.8%; F1 score = 98.3%. (2) For SqueezeNet: Sensitivity = 98.3%; Specificity = 98.7%; Precision = 99.3%; Accuracy = 98.4%; F1 score = 98.8%
Tuncer et al. [85]	Turkey	Unnested case-control study	Radiology	Computer lab	Secondary dataset	(1) GitHub.com; (2) Kaggle.com	Total 321 chest X-ray images: 87 COVID-19, 234 normal	Two experimental studies were carried out: 50% training; 50% testing, 80% training; 20% testing	Classification of COVID-19 vs. normal lung on chest X-ray imaging	Diagnosis of COVID-19	N/A	Chest X-ray images	Developed new model	ML; residual exemplars local binary pattern-based feature extraction (ResExLBP) with iterative relief, using five classification methods: (1) decision trees; (2) linear discriminant; (3) KNN; (4) SVM; (5) subspace discriminant	Higher performance was reported for the training-to-testing ratio of 80:20: (1) Decision tree: Sensitivity = 82.9%; Specificity = 94.4%; Accuracy = 91.3%; balanced classification accuracy = 99.7%. (2) Linear discriminant: Sensitivity = 95.0%; Specificity = 99.9%; Accuracy = 98.6%; balanced classification accuracy = 97.5%. (3) KNN: Sensitivity = 87.6%; Specificity = 100%; Accuracy = 96.6%; balanced classification accuracy = 93.8%. (4) SVM: Sensitivity = 98.0%; Specificity = 100%; Accuracy = 99.4%; balanced classification accuracy = 99.0%. (5) Subspace discriminant: Sensitivity = 93.0%; Specificity = 99.9%; Accuracy = 98.1%; balanced classification accuracy = 96.5%
Ucar et al. [86]	Turkey	Unnested case-control study	Radiology	Computer lab	Secondary dataset	(1) Joseph Cohen dataset; (2) Kaggle.com	Total 5949 X-ray images: 66 COVID-19, 3895 non-COVID pneumonia, 1349 normal	89% training (5310), 11% testing (639)	To provide a rapid diagnostic system able to classify visual properties on COVID-19 X-ray images	Diagnosis of COVID-19	N/A	Chest X-ray images	New model based on existing backbone	ML; DL; CNN based on the pretrained SqueezeNet model with Bayes optimization (COVIDiagnosis-Net)	Specificity = 99.7%; Accuracy = 100%; F1 score = 99.7%; Correctness = 99.3%; Completeness = 100%
Vaid et al. [87]	Canada	Unnested case-control study	Radiology	Computer lab	Secondary dataset	(1) Joseph Cohen dataset; (2) ChestX-ray8 data by Wang et al. 2017	Total 545 chest X-ray images: 181 COVID-19, 364 normal	64% training, 16% validation, 20% testing	To improve the accuracy of COVID-19 reported cases and to precisely predict the disease from chest X-ray scans	Diagnosis of COVID-19	N/A	Chest X-ray images	New model based on existing backbone	ML; DL; CNN based on pretrained VGG-19 with added multilayer perceptron	Sensitivity = 97.1%; Specificity = 96.0%; Precision = 91.7%; Recall = 97.1%; F1 score = 94.3%
Wang et al. [88]	China	Unnested case-control study	Radiology	Hospital-based	Primary dataset	(1) Renmin Hospital of Wuhan University; (2) The First Affiliated Hospital of Anhui Medical University; (3) Beijing Youan Hospital of Capital Medical University; (4) Huangshi Central Hospital; (5) The First Hospital of China Medical City; (6) Henan Provincial People's Hospital; (7) West China Hospital of Sichuan University	Total 1266 patients: 924 COVID-19 pneumonia, 271 bacterial pneumonia, 31 mycoplasma pneumonia, 11 fungal pneumonia, 4106 lung cancer cases (data for 471 patients was used for prognostic analysis)	56% training (709 patients), 31% validation of diagnostic performance (387), 13% validation of prognostic performance (170)	To classify COVID-19 compared to other viral pneumonia and predict the prognosis of COVID-19 patients based on CT images	Diagnosis of COVID-19	Hospital stay-time	Chest CT images	New model based on existing backbones	ML; DL; two models: (1) Pretrained DenseNet121-FPN for lung segmentation; (2) new COVID-19 net for diagnosis analysis; (3) DL combined with cox-proportional hazard model for predicting prognosis	Performance of validation model for classifying COVID-19 from other pneumonia: Sensitivity = 79.3%; Specificity = 71.4%; Accuracy = 85.0%; F1 score = 90.1%; AUC = 0.86 (95% CI = 0.83, 0.89). Measures for the performance of prognostic model were not reported
Wu et al. [89]	China	Retrospective cohort	Internal medicine	Hospital-based	Primary dataset	(1) Central Hospital of Wuhan; (2) Liege, Belgium; (3) Genoa, Italy; (4) Rome, Italy; (5) Hubei province, outside Wuhan; (6) Other provinces in China; (7) Other hospitals in Wuhan	725 COVID-19 cases	From Central Hospital Wuhan: 33% training (239), 8% validation (60). From other data sources: 60% testing (426)	To assess risk severity and triage for COVID-19 patients at hospital admission based on clinical features	N/A	Onset of severe or critical illness during hospitalization	Age, hospital employment, body temperature, time of onset to admission, lymphocyte proportion, neutrophil proportion, C-reactive protein, LDH, creatine kinase, urea, calcium lesion range score on CT	Developed new model	ML; logistic regression. Four models were used based on covariates selected: Model 1: Controlled for age and hospital employment. Model 2: Controlled for age, hospital employment, body temperature, and time of onset to admission. Model 3: Controlled for age and lesion range score. Model 4: Controlled for age, lymphocyte, C-reactive protein, LDH, creatine kinase, urea, and calcium	Average reported performance for Model 4 on different testing data: Sensitivity = 84.6%; Specificity = 73.7%; Accuracy = 80.1%; AUC = 0.88; PPV = 75.2%; NPV = 85.2%
Wu et al. [90]	China	Unnested case-control study	Radiology	Hospital-based	Primary dataset	(1) Renmin Hospital of Wuhan University; (2) The First Hospital of China Medical University; (3) Beijing Youan Hospital in China	Total 495 patients: 368 COVID-19 cases, 127 other pneumonia (368 COVID pneumonia, 127 other pneumonia)	80% training (395), 10% validation (50), 10% testing (50)	To use CT images for screening patients for COVID-19 pneumonia	Diagnosis of COVID-19 and other pneumonia classification	N/A	Chest CT images	New model based on existing backbone	ML; DL; multi-view fusion deep learning model based on the modification of ResNet50	Sensitivity = 81.1% (95% CI = 68.5-93.7); Specificity = 61.5% (95% CI = 35.1-88.0); Accuracy = 76.0% (95% CI = 75.3-76.7); AUC = 81.9% (95% CI = 67.3-96.5)
Yan et al. [91]	China	Retrospective cohort	Internal medicine	Hospital-based	Primary dataset	Tongji Hospital	Total of 485 pregnant and breastfeeding COVID-19 patients	77% training, 23% testing	To reveal the most crucial biomarkers distinguishing patients at imminent risk, thereby relieving the clinical burden and potentially reducing the mortality rate	N/A	Death	Lactate dehydrogenase, lymphocytes, high-sensitivity C-reactive protein	Developed new model	ML; multi-tree XGBoost model	Precision = 81%; F1 score = 90%; Recall = 100%
Yang et al. [92]	China	Retrospective cohort	Radiology	Hospital-based	Primary dataset	Shanghai Public Health Clinical Center	Total 295 patients: 149 COVID-19 cases, 149 non-COVID patients	46% training (69 COVID-19; 66 non-COVID), 7% validation (10 COVID-19, 10 non-COVID), 24% testing (70 COVID-19, 70 non-COVID)	To detect COVID-19 features on high-resolution CT	Diagnosis of COVID-19	N/A	Chest CT images	Used existing pretrained model	ML; DL; CNN Densley Convolutional Networks (DenseNet)	Sensitivity = 97%; Specificity = 87%; Accuracy = 92%; F1 score = 93%; AUC = 0.98 (95% CI = 0.972-0.995)
Yi et al. [93]	USA	Unnested case-control study	Radiology	Computer lab	Secondary dataset	(1) Radiopaedia; (2) RSNA; (3) SIRM	Total 88 COVID-19 chest X-rays	N/A	To classify COVID-19 on chest X-ray images	Diagnosis of COVID-19	N/A	Chest X-ray images	Used existing pretrained model	ML; DL; a pretrained model previously used for the classification of TB	Sensitivity = 89%
Zhang et al. [94]	China	Unnested case-control study	Radiology	Hospital-based	Primary dataset	China Consortium of Chest CT Image Investigation (CC-CCII)	Total 2246 patients (4695 chest CT images): 752 COVID-19 pneumonia cases, 797 other pneumonia patients, 697 normal patients	N/A	To diagnose COVID-19 pneumonia and differentiate it from other pneumonia, and provide prognosis indicators for patients with COVID-19 using a combination of chest CT and clinical parameters	Diagnosis of COVID-19 and classification from other pneumonia classes	Time from the initial hospital admission to severe or critical illness (defined by death or clinical need for mechanical ventilation or transfer to the ICU)	Chest CT images, respiratory function (oxygen saturation index and respiratory rate), age, body temperature on admission, Tmax liver biochemistry markers (albumin, serum LDH, indirect bilirubin), coagulation markers (thrombin time, activated partial thromboplastin time/APTT, platelet count), electrolyte and acid-base balance (Na+, K+, HCO3- ), markers of inflammation (C-reactive protein, lymphocyte count, neutrophil count)	New model based on existing backbone	For diagnosis: ML; DL; DeepLabv3 For prognosis: Regression model - Light Gradient Boosting Machine (LightGBM)	Performance measures for diagnostic model: Sensitivity = 94.9%; Specificity = 91.1%; Accuracy = 92.5% AUC = 0.98 (95% CI = 0.97, 0.99). For prognostic model: Sensitivity = 86.7%; Specificity = 80.0%; AUC = 0.91 (95% CI = 0.88, 0.94)
